# Advanced Anesthesiology and Perioperative Medicine (AAPM) Fellowship Program in Hamad Medical Corporation during COVID-19 Pandemic: Adapting and Redesigning the Fellowship Program

**DOI:** 10.15694/mep.2021.000005.1

**Published:** 2021-01-11

**Authors:** Muhammad Jaffar Khan, Ahmed Zaghw, Tarek Tageldin, Mohamed Elarref

**Affiliations:** 1 Department of Anesthesia

**Keywords:** Medical Education, Healthcare system, Anesthesiology, Trainees, Wellbeing, Telemedicine, Virtual Learning, coronavirus, COVID-19

## Abstract

This article was migrated. The article was marked as recommended.

Health care systems, as well as graduate medical education and training, have experienced unprecedented disruption due to the COVID-19 pandemic. Many academic medical institutions have adopted innovative strategies, technology, and dramatic transformation to continuously provide education and training to physicians in training while providing utmost and urgent care to the growing number of COVID-19 patients. Furthermore, medical societies have prioritized personal well-being, flexibility, and support for the trainees. Herein, we share the experience, lesson learned, practical guidance, and highlight the challenges faced by the program director and fellows of the advanced anesthesiology fellowship program at Hamad Medical Corporation.

## Introduction

The Novel Coronavirus Disease 2019 which emerged as a cluster of unidentified cases of pneumonia in Wuhan, China ( Lu, Stratton and Tang, 2020; Yu *et al.*, 2020) and later designated as COVID-19 by the World Health Organization, caused by Severe acute respiratory syndrome coronavirus 2 (SARS-CoV-2), has become a worldwide pandemic in a short time ( Sohrabi *et al.*, 2020), affecting countries across the globe, causing substantial morbidity and mortality worldwide. The COVID-19 pandemic has resulted in over 17.6 million confirmed cases and over 650 thousand deaths globally as of now. As of 1st august 2020, Qatar has approximately one hundred and eleven thousand confirmed cases and around one hundred and seventy deaths, making it one of the countries with the lowest mortality rate.

The COVID-19 pandemic has detrimental implications for public institutions, and it has shaken the Healthcare system worldwide. It has resulted in an expansive universal public health issue for patients and their health professionals, particularly for trainee physicians who are providing frontline care. Consequently, particular disruption is evident within the medical education sector with infection control policies mandating social and physical distancing. Anesthesiologists have been battling against the COVID-19 pandemic as experts in Airway, Critical care, and Perioperative medicine ( Zhang *et al.*, 2020). Therefore, most of the academic anesthesiology programs have been providing a major proportion of their trainees for the COVID-19 workforce. Likewise, Fellows-in-training has been no exception. Hands-on medical specialties particularly Anesthesiology have been impacted the most ( Daodu *et al.*, 2020). Being at the frontlines of the medical response, there is a need to keep the balance between meeting urgent patient care demands and the pressure to meet training requirements for the in-training program. Because of this growing concern, our academic medical center rapidly adapted to synchronize organizational employee’s needs with trainee safety, medical education, and well-being and innovative educational tools developed to continue medical education during the pandemic.

## Health care system in Qatar

Qatar has rapidly developed a network of health centers and hospitals around the country in recent years to meet the growing healthcare demands of its people. Hamad Medical Corporation (HMC), a non-profit, government-run, health care system in Qatar, has emerged as a state-of-the-art institution with the most sophisticated and advanced infrastructure, healthcare system, accredited by Joint Commission International (JCI), and highly qualified workforce. It includes twelve specialized hospitals under its banner. Additionally, Hamad Medical Corporation is also an academic institution with affiliations to Weill Cornell Medical College and College of Medicine-Qatar University.

## Anesthesia Residency and Fellowship program in HMC

The Department of Anesthesiology, Perioperative Medicine and Critical Care has been committed to continuous medical education and plays an essential role in educating and training residents and fellows of HMC. Our department offers surgical patients with multidisciplinary perioperative care including life-threatening conditions or unstable, severely ill patients who require close monitoring and, those who potentially require intensive intervention and organ support. As a department, we collaborate with other specialties in Hamad Medical Corporation hospitals to ensure the highest possible levels of care for surgical patients. Our department philosophy is based on comprehensive and rigorous medical education, training, and dedication to providing its patients with the best and most efficient care as an Academic Health System. The Anesthesia Residency Program is accredited by Accreditation Council for Graduate Medical Education International (ACGME-I) and it’s a 5-year training program with one Clinical Base Year (CBY), consisting of around 35 residents. Likewise, it offers post-graduate fellowship programs in Advanced Clinical Anesthesia, Regional Anesthesia, Chronic pain, Neuroanesthesia, Obstetric Anesthesia, and Surgical Intensive Care and currently, it has around 15 fellows-in-training.

## The Advanced Clinical Anesthesia Fellowship

Advanced anesthesiology and perioperative medicine fellowship curriculum focus on wide diverse arrays of anesthesia skills. Our Advanced Anesthesiology and Perioperative Medicine (AAPM) Fellowship Program provide the utmost training for the anesthetist to be expert and well versed in taking care of all complex surgical patients with a life-threatening condition who require close monitoring and care during the perioperative period. The Fellowship is designed for anesthesiologists who desire to obtain more experience in the management of particularly challenging cases in the subspecialties such as Neuroanesthesia, Head and Neck Anesthesia/Advanced Airway management, Thoracic Anesthesia, Bariatric Anesthesia, Vascular anesthesia, Obstetrics anesthesia, and Major abdominal surgeries including transplant surgeries. The fellowship aims to allow fellows to become highly trained and capable anesthetists who can confidently take care of high-risk patients and to perform the state-of-the-art anesthesia techniques and skills. After the fellowship training, fellows become expert consults in managing complex airway with expertise on anesthesia subspecialties and complex surgeries with their implications in difficult airway management. Presently, the program has three fellows-in-training.

## HMC during COVID-19 pandemic: System-Wide Incident Command Committee (SWICC)

Hamad Medical Corporation is one of the most modern and advanced healthcare systems in the world is well placed to curb the COVID-19 pandemic. Hamad Medical Corporation with the assistance of the Ministry of Public Health has established the nation’s COVID-19 Command Center called “System-Wide Incident Command Committee” to support the Healthcare system during a pandemic and provide up to date information and guidance to the patients and healthcare workforce. COVID-19 is an emerging disease, with projections for an escalation in inpatient demand that could surpass the capacity of both the general medical ward and intensive care unit by ten times ( Ferguson *et al.*, 2020). In anticipation of those challenges, the SWICC team has been active in managing the crisis and rapidly expand the capacity of hospital beds with 3012 acute beds and 749 ICU beds to provide urgent care for sick patients.

### SWICC actions and Its impact on Anesthesiologist

Many institutions and policymakers have implemented momentous plans in order to monitor the spread of the COVID-19 pandemic. Concurrently, SWICC has executed exceptional changes to the health care system, including the upgrade of general wards to ICU capacities, the deferral and halting of elective surgeries, and setting up separate COVID-19 treatment facilities away from the general inpatient facilities. On the other hand, oncological procedures, emergencies, and, obstetric procedures are given priority and formulating strategies to redefine the roles of the healthcare workforce including anesthesiologists. Educational activities have all been deferred such as morning journal clubs, resident’s didactic teaching activities, departmental Morbidity and mortality meetings and endorsement of critical cases in the surgical critical care unit has been reduced to the minimum on-call intensivist. Anesthesiologists, including residents and fellows-in-training, are part of these changes. Additionally, the SWICC team has instructed each department to keep the physicians including residents and fellows standby to mobilize the workforce to COVID-19 treatment facilities in times of need.

## Redesigning anaesthesiology in-training program during COVID-19 pandemic

The effect of COVID-19 on graduate medical education and training has been considerable ( Potts, 2020). To ensure the continuity of care and medical education, Anaesthesia Department in HMC has adopted a strategy to maximize learning while employing innovative technologies and new guidelines obtained from international medical academic institutes ( Hall *et al.*, 2020). Under such plan, a portion of in-training anaesthesiologists is assigned to hospital duties (i.e., direct patient care, involved in major surgeries, pre-anesthesia assessments, and doing emergency surgeries) whereas the rest is deployed to designated COVID-19 hospitals for two to four weeks. Frequent rotations between departments and hospitals were reduced in anticipation that such rotations make trainees potential vectors for coronavirus. Physicians in-training who are assigned to COVID-19 treating facility can join their primary department after being tested negative for the virus. By doing so, one could avert a situation in which an entire in-training physicians contract COVID-19 which could be catastrophic. Such strategy and its early implementation help mitigate these challenges. Furthermore, to direct administrative, organizational, and educational roles during a pandemic; a list of priorities has been established ( Table 1). In conclusion, our fellowship program demonstrates greater flexibility in training while ensuring competencies.

**Table 1.** Goals of restructuring in-training program

Continuity of high-quality care for surgical patients
Maintenance of trainees health and well-being
Continuity of medical education through virtual teaching and telemedicine
Encouraging individual research and professional opportunities

### Continuity of high-quality care for surgical patients and clinical training

Advanced anesthesia Fellows are a vital part of providing high-quality care for surgical patients. All major oncological surgeries such as hepatic resection, Whipple’s procedure for pancreatic cancer, upper and lower Gastrointestinal tumor resections, and thoracic surgeries which could not be deferred, continued in the operating theatre while receiving a high standard of care. Fellows are ensured to obtain experience and skills in managing such complex oncological surgeries during the pandemic. Similarly, fellows underwent proper orientation and educational lectures before assigned to the COVID-19 facility which includes appropriate training on hand hygiene, N95 mask fitting, Personal Protective Equipment (Donning and Doffing technique), airway and ventilator management of COVID-19 patients, use of proning technique for the severely hypoxic patient, pharmacology of sedatives, analgesia and paralyzing drugs and other critical-care-focused specialties ( Table 2).

**Table 2.** Summary of a week-long COVID-19 educational series and simulations

Education on global health perspectives on COVID-19 pandemic
Pathophysiology, diagnosis and management of COVID-19, Acute respiratory distress syndrome and associated complications
Simulations on proper personal equipment technique (donning and doffing) and COVID-19 intubation checklist
Airway and ventilatory management of COVID-19 patient
Proning and deproning technique and utilization of sedative and paralytic drugs in critical care unit
Bedside point-of-care ultrasound for COVID-19 associated cardiac and pulmonary complications
Utilization of COVID-19 guidelines on sepsis and advance life support (ALS)
Utilization of educational resources on the management of stress and maintenance of well-being and health

### Maintenance of health and well-being

Foreseeing the drastic psychological impact on trainees during this stressful time, maintenance of trainees’ health and well-being remains a central focus. Trainees have been provided with appropriate awareness tools and resources for the maintenance of optimal mental health. It has been shown that work-related stress has mental health sequelae on healthcare workers who are deployed at COVID-19 facilities ( Talevi *et al.*, 2020; Torales *et al.*, 2020). As a result, it may impede a physician’s decision-making capacity and have an enduring impact on their wellbeing ( Kang *et al.*, 2020). Before deployment to COVID-19 facilities, the Trainees’ wellbeing questionnaire was circulated to all trainees including residents and fellows to identify trainees at risk of COVID-19 infection and its complications. The duty rota is modified in a way to keep some trainees standby from home to reduce the risk of exposure and are given ample hours per week for rest. Trainees are being assured of the availability of Personal Protective Equipment all the time and have been given adequate training to perform clinical duties properly in COVID-19 facilities. Furthermore, Physician In-training has been provided with alternative accommodation when necessary to avoid transmitting the disease to the loved ones at home. Also, the healthcare providers working in the COVID-19 facility receive monetary compensation for overtime and Free breakfast and lunch boxes are distributed for the trainees. COVID-19 psychological support hotline for healthcare professionals has launched. The Government and the corporation ensured the continuation of funds and salaries to the trainees during the pandemic. Finally, to keep the trainees and healthcare workers informed and updated, a single concise message and information is sent daily by the SWICC team.

### Telemedicine and Telelearning

In anticipation of the risk of transmitting the disease to other patients especially vulnerable patients and healthcare providers, many medical centers initiated telemedicine technologies around the globe. Simultaneously, our institutions adopted the same strategies where outpatient clinics and healthcare centers are encouraged to practice telemedicine. Common hotline number established to facilitate the process and continuity of healthcare for outpatients. Physicians and healthcare workers have access to the patients’ charts electronically via Cerner (electronic health record technologies) and hence, can monitor the patients remotely while reducing the risk of coronavirus exposure. As in-person teachings and meetings are postponed due to the pandemics, many in-training programs resort to virtual teaching and tele-education, encourage self-directed learning and problem-based learning via teleconferencing. Web-based online platform technologies are utilized to continue medical education and can even be interactive. For example, the Usage of Microsoft teams application ( Microsoft, 2020) by logging via institutional email for free, which is an integrated virtual forum for online conferences and collaboration through Microsoft applications (Word, PowerPoint, and Excel). Also, it is compatible with iOS, Android, Microsoft surface, web browser. In-training and faculty members employ this technology ( Wang, Deng and Tsui, 2020) for the continuation of medical education such as didactic lectures, journal clubs, case, and problem-based discussions. Likewise, video lectures and other educational resources particularly related to COVID-19 can be easily uploaded and accessed by the in-training physicians. Our program anticipates a growing role for online education to augment conventional teaching allowing for greater versatility in this new era of free access to online medical resources and technology-enhanced learning ( Wang, Deng and Tsui, 2020).

### Research and professional opportunities

Trainees are encouraged to take part in any ongoing research and quality improvement project especially related to COVID-19. As the elective surgical cases reduced, and rota arranged in a way to keep trainees stand-by and away from the hospital environment, it has provided ample opportunities to delve into research, quality improvement projects, audits, and other professional opportunities. The program director and faculty members impart a great deal of guidance and teaching to their trainees related to research and developing a professional career during a pandemic.

## Joining Qatar’s ICU-COVID 19 workforce: Advanced Clinical Anaesthesia Fellow’s Perspective

The COVID-19 Pandemic has posed many challenges for Anesthesia and Critical care and it has upset the Healthcare structure universally, with staff often working outside their comfort zones, staffing and logistics issues, and work-related anxieties about the provision of personal protective equipment. However, Healthcare worker’s morale has been high ever since.

Anesthetist’s role is changing, and increasingly they are asked to join the ICU workforce ( Ortega and Chen, 2020). COVID-19 challenges one’s adaptability, flexibility, resilience, and responsiveness to problems. Fortunately, the Advanced anesthesia fellows work in a dynamic and progressive department where many plans and strategies are made and implemented. Our Critical care units have been under extreme pressure and most commonly trainees have been asked to join the ICU-COVID 19 workforce.

Advanced anesthesia fellows have been playing their role in providing care to critically ill patients across HMC’s COVID-19 facilities. Their roles as front line healthcare providers including but not limited to airway management, invasive lines insertion, procedural sedation, escorting a critically ill patient to the imaging facility, managing RRT (rapid response team), and code blue ( Table 3). They are extremely well-supported and have built an incredible understanding with intensivists. Working in critical care units under extraordinary circumstances offers unique challenges where healthcare workers and trainee physicians are working outside their comfort zones, concerns related to the shortage of PPE, and fears of transmitting the disease to the loved ones. However, the trainees continue to show resilience, immense compassion, selflessness, and dedication during the pandemic.

**Table 3.** Sample ofdaily procedures performed by advanced anesthesia fellows during 12 hours’ shift in ICU-COVID-19 facility

Type of procedures	Number of procedures
Airway management	4
Arterial line insertion	6
Central line/ Dialysis catheter insertion	5
Rapid response team/code blue	4

## Conclusion

The COVID-19 pandemic has imposed extraordinary challenges and disruption of medical education as healthcare systems continue to stretch with an additional unprecedented burden on critical care medicine services. Hence, it necessitates a paradigm shift from conventional medical education and the rapid implementation of contingency plans. Controlling the spread of COVID-19 has become the essential focus of almost all countries worldwide. During COVID-19 and emergency plans, fellows’ wellbeing and education are the top priority. The fellowship training has greater flexibility to accommodate their needs. Academic medical centers must keep a balance between meeting the demands of the healthcare workforce and the continuity of clinical education and training. As trainees are well suited to lead during the pandemic; academic medical institutions must redesign the in-training program to sustain clinical knowledge, skills, and research while ensuring the utmost attention to trainee’s health and wellbeing. These strategies may guide other specialties on how to implement in-training program restructuring while perpetuating a core set of values.

## Take Home Messages

• The COVID-19 has emerged as a great challenge for the continuation of postgraduate medical education
• There is a need for new innovative alternative techniques for educating and training medical trainees while maintaining course objectives
• Residency and Fellowship training program must show flexibility to meet the demands of its trainees
• It is important to maintain a balance between meeting the demands for the healthcare workforce and the continuity of medical education and trainee’s wellbeing.

## Notes On Contributors

**Muhammad Jaffar Khan, MBBS** is a clinical fellow in Advanced Anesthesia and Perioperative Medicine in the Department of Anesthesia, Critical Care Medicine and Perioperative Medicine at Hamad Medical Corporation, Qatar. He also completed his residency in anesthesiology in the same institution. Dr. Khan graduated from West China School of Medicine, Sichuan University, PRC. His research interests include medical education, resuscitation in trauma and burns, and advanced airway.

ORCiD: https://orcid.org/0000-0001-5082-5906

**Ahmed Zaghw, MD** is a medical graduate from Ain-Shams University, Cairo, Egypt. He completed his residency in anesthesiology in the Department of Anesthesia, Critical Care and Perioperative Medicine. He is currently doing fellowship in Advanced anesthesia and Perioperative medicine in the same institution. His research interests include medical education and informatics, advanced airway management, thoracic and transplant anesthesia.

ORCiD: https://orcid.org/0000-0003-0316-488X

**Tarek Tageldin, MD** graduated from faculty of medicine, Alexandria University, Egypt. He completed his residency training in Anesthesiology and currently doing his clinical fellowship in Advanced Anesthesia and Perioperative Medicine in the Department of Anesthesia, Critical Care Medicine and Perioperative Medicine at Hamad Medical Corporation, Qatar. His research interests include advanced airway, bariatric, robotic and transplant anesthesia.

**Mohamed Abdelwahab Mohamed Elarref, MBChB, MSc, MD** is a senior consultant and fellowship program director of Advanced anesthesia and Perioperative Medicine in Department of Anesthesia, Critical care and Perioperative medicine in Hamad medical corporation, Qatar. He is also Assistant Professor at Weill Cornell Medicine - Qatar. He graduated from Faculty of medicine, Tanta University, Egypt. He joined the department with special interest in educating medical students and residents. His research interests include medical education, perioperative medicine, advanced airway and regional anesthesia.

ORCiD: https://orcid.org/0000-0002-3865-2667
