# Supplementary material for: Advanced Anesthesiology and Perioperative Medicine (AAPM) Fellowship Program in Hamad Medical Corporation during COVID-19 Pandemic: Adapting and Redesigning the Fellowship Program
Source: MedEdPublish (2016). 2021 Jan 11;10:5. [Version 1] doi: 10.15694/mep.2021.000005.1 (PMC10939612; doi:10.15694/mep.2021.000005.1)
Supplement: Peer review history

# Reviewer report: Reviewer response for version 1

**DOI:** 10.21956/mep.18862.r26903

**Published:** 2021-06-01

**Authors:** Ken Masters

**Affiliations:** 1 Sultan Qaboos University

This review has been migrated. The reviewer awarded 3 stars out of 5 An interesting paper dealing with the adaptation of a Fellowship Program because of the COVID-19 pandemic.The paper gives good insight into the impact of COVID-19 and the health services situation in Qatar and the specific centre. Although the paper is focused on one country, and is not intended to be generalisable, it does help to complete a world picture of how academic medical institutions have adapted to the crisis.Some points (mostly minor) that need addressing:• There are quite a few language and punctuation errors, and a careful proof-read would benefit the paper. (Mostly, these errors do not impede understanding, but they do become an irritation).• “Hamad Medical Corporation is one of the most modern and advanced healthcare systems in the world is well placed to curb the COVID-19 pandemic”. This sentence should end with “..in Qatar”, otherwise it may be read to imply a wider impact. (If that wider impact is intended, that it would be useful to have it stated explicitly, so that there is no room for misunderstanding.)• “Before deployment to COVID-19 facilities, the Trainees’ wellbeing questionnaire was circulated to all trainees including residents and fellows to identify trainees at risk of COVID-19 infection and its complications.” This was a good step, but the paper should describe what happened as a result of this exercise.• Some of the paragraphs are quite lengthy and could benefit from being broken into smaller pieces.So, a useful overview. I look forward to Version 2 of the paper in which these issues, especially the language problems, are addressed.

Reviewer Expertise:

NA

No decision status is available

# Reviewer report: Reviewer response for version 1

**DOI:** 10.21956/mep.18862.r26902

**Published:** 2021-01-19

**Authors:** Balakrishnan Nair

**Affiliations:** 1 Centre for Medical Professional Development and University of Newcastle

This review has been migrated. The reviewer awarded 4 stars out of 5 Thanks for asking me to review this I enjoyed reading this article. What the team has done is innovative, even though this term had been overused during the COVID pandemic in other contexts. Their innovation included setting up the System based Incident Command Committee to oversee the patient care during the crisis. The project has maximised the learning needs whilst innovating care of their patients. The staff were re-deployed, upskilled whilst protecting the trainees' health care and learning needs. Targetted education was provided rather than throwing the trainee in at the deep end. Overall an excellent project and the readership can learn from this. Some evaluation of the learning and outcomes would have been useful.

Reviewer Expertise:

NA

No decision status is available
